# Access to Dermatological Appointments Based on Insurance Types in Hawaiʻi

**DOI:** 10.7759/cureus.66650

**Published:** 2024-08-11

**Authors:** Amity Tran, Emily Leibovitch, Michelle Stafford, Devashri Prabhudesai, John J Chen, Nash Witten

**Affiliations:** 1 Internal Medicine, University of Hawaiʻi at Mānoa, John A. Burns School of Medicine, Honolulu, USA; 2 Pediatrics, University of Hawaiʻi at Mānoa, John A. Burns School of Medicine, Honolulu, USA; 3 Psychiatry, University of Hawaiʻi at Mānoa, John A. Burns School of Medicine, Honolulu, USA; 4 Quantitative Health Sciences, University of Hawaiʻi at Mānoa, John A. Burns School of Medicine, Honolulu, USA; 5 Family Medicine and Community Health, University of Hawaiʻi at Mānoa, John A. Burns School of Medicine, Honolulu, USA

**Keywords:** hawaiʻi, dermatology, medicare, medicaid, disparities, insurance

## Abstract

Although there is evidence that Medicaid beneficiaries in the continental United States experience barriers to accessing dermatological care, limited data exists on whether these same barriers exist in Hawaiʻi. Using a secret shopper study design, a total of 46 dermatology offices were contacted, 41 (89%) of which were accepting new patients. Thirty (73%) offices were located on Oʻahu, and the remaining 11 (27%) were distributed among the neighboring islands (Hawaiʻi Island, Kauaʻi, Maui). Overall, the acceptance rate for Medicaid (n=14) was 34%, which was significantly lower (P<.0001) than private insurance (n=39 (95%)) and Medicare (n=38 (93%)). The acceptance rate for patients with Medicaid insurance was lower for Oʻahu offices (27%) than for neighboring islands' offices (55%), but the difference was not statistically significant (P=.095). Differences in average wait times were not statistically significant among insurance types or between Oʻahu and neighboring islands. Overall, these results suggest that Medicaid recipients compared to those with private insurance or Medicare might experience difficulty in accessing dermatological care in Hawaiʻi.

## Introduction

Studies conducted in the continental United States have revealed that Medicaid recipients experience substantially lower acceptance rates and longer wait times for dermatological care than patients with private insurance or Medicare [1,2]. These barriers to care have been attributed to numerous factors such as insurance type, geography, and low reimbursement rates [1-3]. Unfortunately, this problem of access to dermatology appointments has also affected the Medicaid population of Hawaiʻi. A 2015 study found that 65% of Hawaiʻi dermatologists would not accept new Medicaid patients [4]. Additionally, since late 2013, with the first Marketplace Open Enrollment Period and Medicaid program changes, there has been a 45% increase in Hawaiʻi's Medicaid population, with a 39% increase since the start of the COVID-19 pandemic in 2020 [5,6]. With this dramatic increase in Medicaid enrollment statewide since 2013 and the 2015 study [4] showing only a 65% acceptance rate for Medicaid, an update on the acceptance rate of Medicaid patients at dermatology practices is needed to better understand the current dermatological healthcare needs of the state of Hawaiʻi. The goal of this study is to update the understanding of the current dermatological healthcare needs of Medicaid patients by analyzing insurance acceptance rates among dermatologists and to determine if appointment wait times vary based on insurance type and island location.

This article was previously presented as an oral presentation at the 2022 Annual Biomedical Sciences Symposium on April 7, 2022.

## Materials and methods

A list of practicing dermatologists in Hawaiʻi was obtained from the American Academy of Dermatology directory [7] and the US Centers for Medicare and Medicaid Services National Provider Identifier list [8]. This was done by using the websites' search filters to provide results for dermatologists listed as practicing in the state of Hawaiʻi. During this initial search, the island where the clinic was located was also recorded. The research team placed scripted telephone calls to each dermatology office thrice to request a self-referred, new-patient appointment for a changing mole on a fictitious adult patient. The three calls differed by insurance type: private insurance (Hawaii Medical Service Association Preferred Provider Organization (HMSA PPO)), Medicare, or Medicaid (HMSA Quality care, Universal access, Efficient utilization, Stabilizing costs, and to Transform (QUEST)). The fictitious patient in this project utilized health insurance provided by HMSA as this is the largest health provider network in the state of Hawaiʻi. Before gathering data, researchers were instructed to set the scene by saying they wanted to schedule an appointment for the evaluation of a changing mole. During the call, the research team collected data regarding whether or not the clinic was accepting new patients, insurance acceptance, the number of days until the first available appointment, and the type of clinician that was assigned for this first available appointment (MD, DO, PA, or NP). If the clinician that was assigned was a PA or NP, the caller would then gather data regarding how many days it would be until an appointment with an MD or DO was available. Prior to ending the call, callers ensured that no official appointment was made. Group practices, where there were multiple dermatologists practicing one clinic, were called once unless there were unique numbers for different partners listed in the directories searched. Clinics not accepting new patients, those only serving military personnel (e.g., Tripler Army Medical Center), or patients solely within their clinic system (e.g., Kaiser Permanente clinics) were excluded from the study and not included in the data analysis. Only clinics still in operation and offering medical dermatology services to new adult patients were included in the study. The data was then de-identified and analyzed by members of the Biostatistics Core. Count and percentages of new patient acceptance were summarized, overall and by key variables, e.g., insurance type, island, and days until the first available appointment. McNemar's test was performed to evaluate insurance acceptance differences. Mean wait times for different insurance types and different islands were calculated; the mean wait time for Medicaid was compared against the mean wait times for Medicare and private insurance using the paired t-test. The mean wait times for all insurance types were compared using repeated measures analysis of variance (ANOVA). Statistical analyses were performed using R version 4.0.2 (Vienna, Austria). The University of Hawaiʻi Institutional Review Board reviewed this study (IRB ID 2021-00683) and determined it to be not human subjects research.

## Results

**Figure 1:**
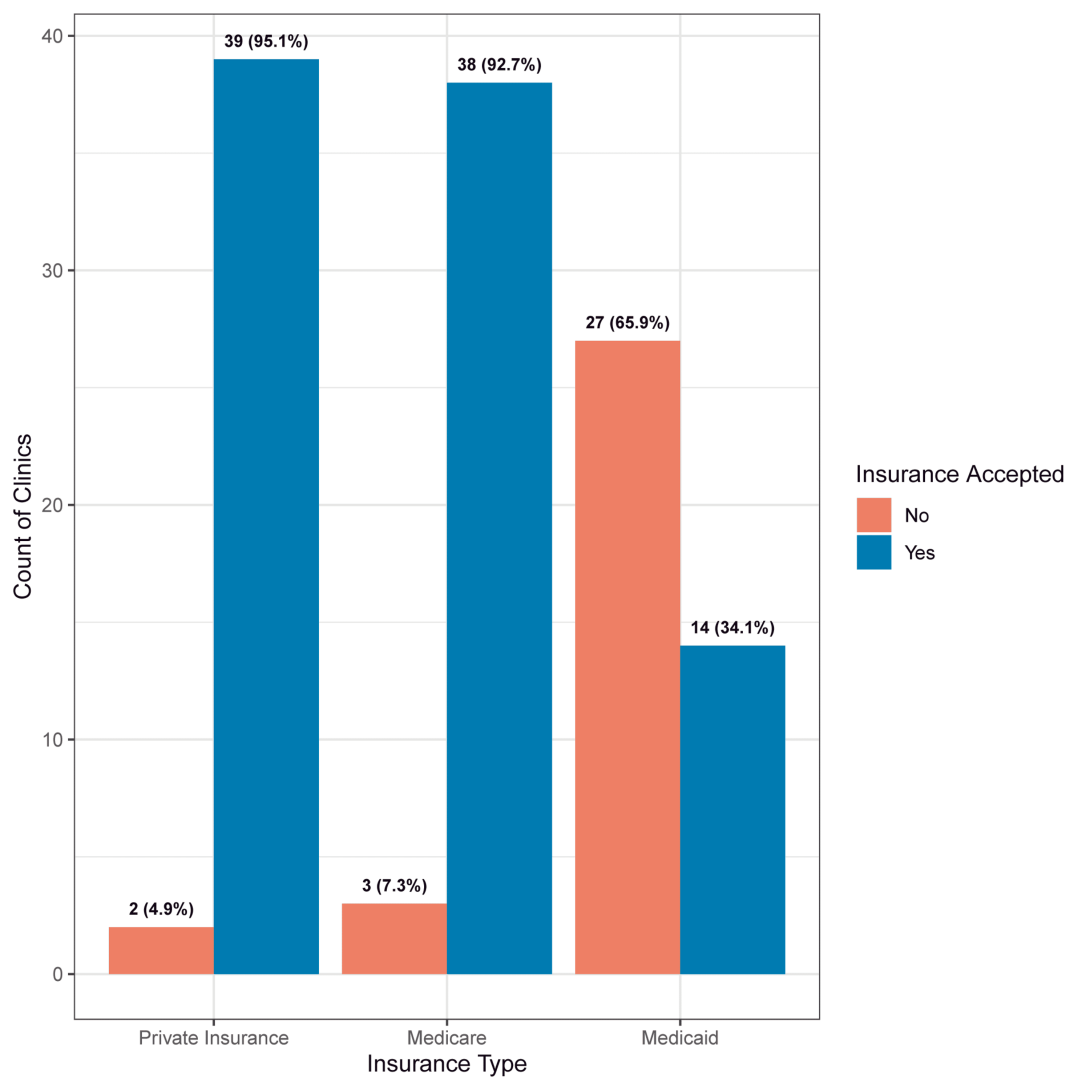
Overall insurance acceptance rates for dermatology clinics accepting new patients (n=41) in the state of Hawaiʻi in 2022.

**Figure 2:**
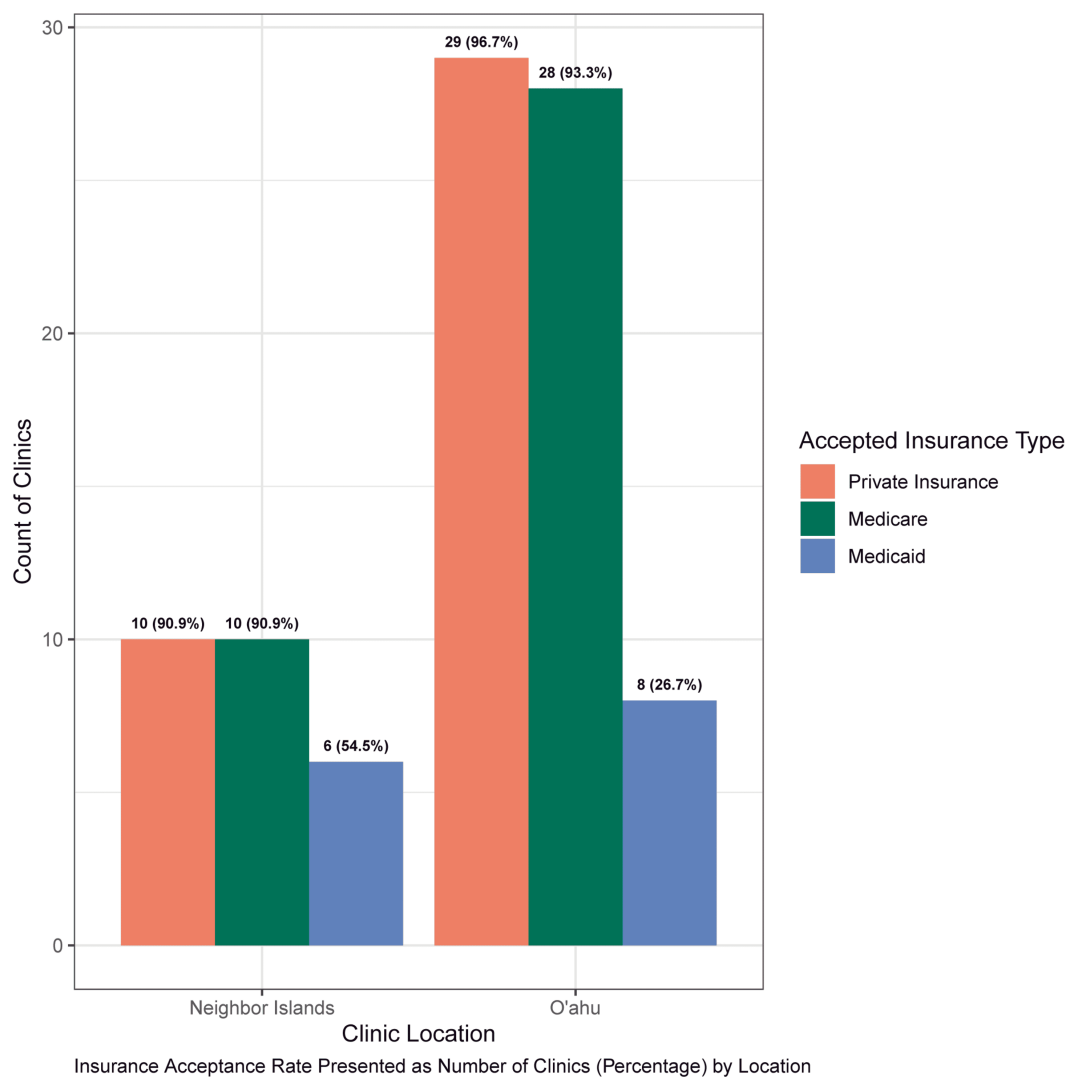
Insurance acceptance rates between dermatology clinics on Oʻahu (n=30) and neighboring islands (Hawaiʻi Island, Kauaʻi, Maui) (n=11) in 2022.

Forty-six of the 58 dermatology practices identified met the inclusion criteria. Of these 46 practices, 41 (89%) accepted new patients. Thirty (73%) offices were located on Oʻahu, and the remaining 11 (27%) were distributed among the neighboring islands (Hawaiʻi Island, Kauaʻi, Maui). Presented in Figure 1 and Table 1 is the acceptance rate for Medicaid (n=14) at 34%, which was significantly lower (P<.0001) compared to private insurance (n=39 (95%)) and Medicare (n=38 (93%)). Two of the offices were out-of-network with all insurance plans, instead offering services for an out-of-pocket cost. The acceptance rate for patients with Medicaid insurance was lower for Oʻahu offices (27%), compared with offices on the neighboring islands (55%), but the difference was not statistically significant (Figure 2). The mean wait time for an appointment with the next available practitioner, e.g., nurse practitioners and physician associates, for patients with Medicaid (46.7 days (SD: 46.4; IQR: 70.8)) was higher than for patients with private insurance (37.9 days (SD: 39.7; IQR: 33.2)) or Medicare (42.2 days (SD: 46.4; IQR: 48)), but the differences were not statistically significant (Table 2). The mean wait times for an appointment with an MD or a DO were similar for Medicaid (49.9 days (SD: 45.2; IQR: 69.8)) and Medicare (49.4 days (SD: 43.1; IQR: 42)) while smaller for private insurance (42.0 days (SD: 39.1; IQR: 43)), and the differences were not statistically significant (Table 2). No significant difference was found in the wait times between Oʻahu and neighboring islands.

**Table 1:** Number of dermatologists in Hawaiʻi accepting new patients with Medicaid insurance in 2022.

Clinic location	Medicaid acceptance	
	Accepts	Doesn't accept
Oʻahu	8 (27%)	22 (73%)
Neighboring islands	6 (55%)	5 (45%)
Total	14	27

**Table 2:** Comparison of mean wait times for the next available appointment between type of insurance and practitioner in 2022. Repeated measures ANOVA showed no significant difference between wait times between the insurance times. ANOVA: analysis of variance; SD: standard deviation; IQR: interquartile range; MD: medical doctor; DO: doctor of osteopathic medicine

Type of practitioner	Type of insurance		
	Medicaid mean days (SD, IQR)	Medicare mean days (SD, IQR)	Private mean days (SD, IQR)
Mid-level practitioner	46.7 (46.4, 70.8)	42.2 (46.4, 48)	37.9 (39.7, 33.2)
Physician (MD or DO)	49.9 (45.2, 69.8)	49.4 (43.1, 42)	42 (39.1, 43)

## Discussion

Overall, these results suggest that Medicaid recipients compared to those with private insurance or Medicare continue to experience difficulty in accessing dermatological care in Hawaiʻi due to limited availability of providers. While there was no significant difference in wait times for Medicaid patients, sifting through physician directories to successfully secure an appointment with dermatologists accepting Medicaid can be time-consuming. Given that the patient in our script had a changing mole, prolonging the time it takes for a Medicaid patient to find a dermatologist may negatively affect those with a serious skin disease by impeding their accessibility to care. These obstacles can result in a later diagnosis, delays in surgical treatment, and poor dermatological health outcomes, which prior studies have shown to be associated with Medicaid and low socioeconomic status [9-11]. This study's findings also demonstrate that Hawaiʻi Medicaid patients may be at a disadvantage when it comes to accessing appointments for skin cancer screenings due to prolonged wait times and a lack of QUEST participating providers, which has also been identified as a significant contributor to the inequities seen in dermatological care [12].

Depending on the island of residence, difficulties accessing dermatological healthcare may be further exacerbated due to the lack of availability of dermatology clinics. The neighboring islands (Hawaiʻi Island, Kauaʻi, Maui) combined only had 11 clinics accepting new patients, while Oʻahu alone had 30 clinics accepting new patients. The islands not included in this study (Molokaʻi, Lānaʻi, Niʻihau) do not have dermatology clinics. Neighboring island Medicaid patients seeking specialized dermatological care may also experience additional financial and administrative burdens that those on Oʻahu do not have. For example, neighboring island patients must coordinate with their provider and Med-QUEST to have their medical travel request approved for coverage. If their travel request is not approved, patients will have to decide between paying for their own travel arrangements and paying out-of-pocket for care on their home island [4].

Although the motivations underlying a clinic's decision not to accept Medicaid may not be derived from the current study, existing data suggests that low reimbursement rate and administrative burden may be significant factors [1,4,13,14]. However, further research is needed to understand the motives behind why the majority of dermatology clinics in Hawaiʻi reject care to patients with Medicaid insurance.

Compared to the previous 2015 study [4], this study showed that the number of dermatologists accepting Medicaid has not changed much, from 35% to 34%, despite the large increase in Medicaid enrollment during the same time. This is concerning because the number of skin cancer cases in Hawaiʻi has increased by 14% from 2015 to 2019 [15]. This study indicates that access to dermatological care has not improved, and has likely worsened, since the 2015 study [4] due to the increased Medicaid enrollment statewide, yet there is a similar rate of acceptance of this insurance type by dermatologists. These findings are crucial to be able to appropriately advocate for improved access to dermatological services in Hawaiʻi for the larger cohort of Medicaid patients. However, until it is known why most clinics choose not to accept Medicaid, the appropriate policy changes cannot be made, leaving the Medicaid population critically underserved in areas of dermatological care.

Our study has several limitations. In our script, we asked about acceptance for HMSA QUEST. We did not include other QUEST providers, such as UnitedHealthcare, AlohaCare, and Ohana. Some clinics may deny QUEST from HMSA while accepting QUEST from other providers, potentially yielding a different result than the one obtained from our study. Additionally, the calls were placed throughout November and early December, so the wait times could have been affected by the holiday season.

## Conclusions

The study found that acceptance rates for Medicaid among Hawaiʻi dermatologists were significantly lower than private insurance and Medicare. Although the average wait time between all insurance types was not significant, Medicaid recipients, especially those who reside on neighboring islands, still experience limited access to dermatological care. By not accepting Medicaid patients, the majority of Hawaiʻi dermatologists are putting Medicaid patients at a higher risk of experiencing poor dermatological health outcomes and unnecessary financial and administrative burdens. Our study suggests that improving Medicaid participation among Hawaiʻi dermatologists is needed to expand equitable access to dermatological care for Medicaid recipients, especially with the increase in the total number of Medicaid patients statewide. Future research into the particular reasons why Hawaiʻi's dermatologists are not accepting Medicaid patients is needed to better advocate for policy changes to improve access to dermatological services.

## References

[1] Resneck J Jr, Pletcher MJ, Lozano N (2004). Medicare, Medicaid, and access to dermatologists: the effect of patient insurance on appointment access and wait times. J Am Acad Dermatol.

[2] Creadore A, Desai S, Li SJ (2021). Insurance acceptance, appointment wait time, and dermatologist access across practice types in the US. JAMA Dermatol.

[3] Resneck JS Jr, Isenstein A, Kimball AB (2006). Few Medicaid and uninsured patients are accessing dermatologists. J Am Acad Dermatol.

[4] Ferrara ML, Johnson DW, Elpern DJ (2015). Cherry picking in the ‘Aina: inequalities of access to dermatologic care in Hawai‘i. Hawaii J Med Public Health.

[5] Medicaid & CHIP in Hawaii (2022). Medicaid & CHIP in Hawaii. https://www.medicaid.gov/state-overviews/stateprofile.html?state=hawaii.

[6] (2022). Hawaii Medicaid enrollment report (2022). https://medquest.hawaii.gov/content/dam/formsanddocuments/resources/enrollment-reports/Website%20Enrollment%20Report%202022%2020220913.pdf.

[7] (2022). Find a dermatologist. https://find-a-derm.aad.org.

[8] (2022). NPPES NPI registry. https://npiregistry.cms.hhs.gov/search.

[9] Amini A, Rusthoven CG, Waxweiler TV, Jones BL, Fisher CM, Karam SD, Raben D (2016). Association of health insurance with outcomes in adults ages 18 to 64 years with melanoma in the United States. J Am Acad Dermatol.

[10] Zell JA, Cinar P, Mobasher M, Ziogas A, Meyskens FL Jr, Anton-Culver H (2008). Survival for patients with invasive cutaneous melanoma among ethnic groups: the effects of socioeconomic status and treatment. J Clin Oncol.

[11] Adamson AS, Zhou L, Baggett CD, Thomas NE, Meyer AM (2017). Association of delays in surgery for melanoma with insurance type. JAMA Dermatol.

[12] Cortez JL, Fadadu RP, Konda S, Grimes B, Wei ML (2022). Disparities in access for melanoma screening by region, specialty, and insurance: a cross-sectional audit study. JAAD Int.

[13] Perloff JD, Kletke P, Fossett JW (1995). Which physicians limit their Medicaid participation, and why. Health Serv Res.

[14] Sommers AS, Paradise J, Miller C (2011). Physician willingness and resources to serve more Medicaid patients: perspectives from primary care physicians. Medicare Medicaid Res Rev.

[15] (2022). Cancer statistics at a glance. https://gis.cdc.gov/Cancer/USCS/?CDC_AA_refVal=https%3A%2F%2Fwww.cdc.gov%2Fcancer%2Fdataviz%2Findex.htm#/AtAGlance/.

